# Allopolyploidy and the evolution of plant virus resistance

**DOI:** 10.1186/1471-2148-14-149

**Published:** 2014-07-03

**Authors:** John Gottula, Ramsey Lewis, Seiya Saito, Marc Fuchs

**Affiliations:** 1 Department of Plant Pathology and Plant-Microbe Biology, Cornell University, New York State Agricultural Experiment Station, Geneva, NY 14456, USA; 2 Department of Crop Science, North Carolina State University, 4310 Williams Hall Campus, Box 7620, Raleigh, NC 27695-7620, USA; 3 Kearney Agricultural Research Center, University of California, Parlier, CA 93648, USA

**Keywords:** Allopolyploidy, *Grapevine fanleaf virus* (GFLV), Nepovirus, Nicotiana, Resistance, Susceptibility, *Tomato ringspot virus (ToRSV)*

## Abstract

**Background:**

The relationship between allopolyploidy and plant virus resistance is poorly understood. To determine the relationship of plant evolutionary history and basal virus resistance, a panel of *Nicotiana* species from diverse geographic regions and ploidy levels was assessed for resistance to non-coevolved viruses from the genus *Nepovirus*, family *Secoviridae*. The heritability of resistance was tested in a panel of synthetic allopolyploids. Leaves of different positions on each inoculated plant were tested for virus presence and a subset of plants was re-inoculated and assessed for systemic recovery.

**Results:**

Depending on the host-virus combination, plants displayed immunity, susceptibility or intermediate levels of resistance. Synthetic allopolyploids showed an incompletely dominant resistance phenotype and manifested systemic recovery. Plant ploidy was weakly negatively correlated with virus resistance in *Nicotiana* species, but this trend did not hold when synthetic allopolyploids were taken into account. Furthermore, a relationship between resistance and geographical origin was observed.

**Conclusion:**

The gradients of resistance and virulence corresponded to a modified matching allele model of resistance. Intermediate resistance responses of allopolyploids corresponded with a model of multi-allelic additive resistance. The variable virus resistance of extant allopolyploids suggested that selection-based mechanisms surpass ploidy with respect to evolution of basal resistance to viruses.

## Background

The ‘Red Queen Hypothesis’ suggests that coevolution between hosts and pathogens or pests results in a ‘boom and bust’ cycle where neither host nor its invader can gain lasting supremacy [1]. Allopolyploidy could provide an opportunity for host species to outpace Red Queen coevolution and achieve epochal gains in resistance such as when two moderately-resistant diploids give rise to an allotetraploid with a full complement of resistance genes. This allopolyploid resistance hypothesis incorporates resistance into models explaining heterosis [2,3], and has been tested experimentally in multiple plant and animal systems [4,5]. Allopolyploidization contributes to 2-4% of speciation events in Angiosperms [6].

Viruses have challenged plants for millennia [7-9]. The genus *Nicotiana* has been used as a model system for studying plant-virus interactions and for investigating genotypic and phenotypic changes that occur at and after polyploidization [10]. The genus *Nicotiana* has 76 recognized species, 35 of which are allotetraploids arising from at least five independent interspecific allopolyploidization events [10]. The most likely diploid progenitors of most *Nicotiana* allopolyploids have been determined using nuclear and plastid DNA sequence information [11-14]. While the majority of *Nicotiana* allopolyploids retained their original chromosome number, most species in section *Suaveolentes* underwent a reduction in chromosome number. Genomic changes can occur in the earliest generations following polyploidization [15-18], and all well-studied *Nicotiana* allotetraploids have undergone gene loss or conversion [12,13]. The main center of diversity for *Nicotiana* is Bolivia and the natural range of this genus extends throughout South America, to the Western US, Australia and Africa [10]. In particular, *N. tabacum* and *N. rustica* likely originated in South America, *N. clevelandii* and *N. quadrivalvis* are endemic to the Western US, and all but one species of section *Suaveolentes* are endemic to Australia [10].

Plant viruses are commonly characterized by their experimental host ranges, sometimes incorporating reactions on *Nicotiana* species in their descriptions [19]. The susceptibility status of *N. tabacum* is known for 541 plant viruses, and at least 29 *Nicotiana* species have been used in virus host range studies [20]. Members of *Nicotiana* section *Suaveolentes* (such as *N. benthamiana*) tend to have the widest experimental host ranges [21-23] and *N. benthamiana*’s multi-pathogen susceptibility makes it an important tool for phytopathology research [10,24]. Although the biological basis of *Nicotiana* nonhost resistance to viruses is unknown, a mutated form of *RNA-dependent RNA polymerase 1* in *N. benthamiana* compromises its broad-spectrum antiviral resistance response [25]. Several dominant, strain-specific virus resistance mechanisms have been described in *Nicotiana*[26-28], and closely related viruses exhibit differential capacities for *Nicotiana* systemic infection [27,28].

Interspecific hybridization can be a useful tool for transferring resistance genes to crops species and for investigating virus resistance [19,29,30]. Interspecific (euploid) hybrids of *Solanum tuberosum* and *S. brevidens* showed quantitative resistance to three diverse potato viruses compared to *S. tuberosum,* which exhibited high virus titers after inoculation [31]. The broad-spectrum virus resistance was quantitatively enhanced if the hybrid contained additional copies of the *S. brevidens* genome or if the plants were aneuploids missing an *S. tuberosum* chromosome [31]. Introgression of an alien chromosome from *N. africana* into *N. tabacum* produced tolerance (an amelioration of symptoms) to *Potato virus Y* in *N. tabacum,* but did not confer the immunity exhibited by *N. africana per se*[32]. These data support the conclusion that basal virus resistance is quantitatively controlled by multiple genes.

Nepoviruses are *ne*matode-transmitted *po*lyhedral-shaped viruses of the family *Secoviridae*[33]. These viruses, including *Grapevine fanleaf virus* (GFLV) and *Tomato ringspot virus* (ToRSV), have single-stranded bipartite RNA genomes in positive-sense orientation. GFLV and ToRSV are present in most arable temperate regions and cause severe economic losses to grapevine and other crops [34,35]. Based on the distribution of their highly specific nematode vectors, the likely origins of GFLV and ToRSV are the Near East and Eastern North America, respectively [36,37]. *N. tabacum* exhibits a recovery reaction after infection of GFLV and ToRSV, and salicylic acid (SA)-based resistance mechanisms appear to be critical for recovery from ToRSV [38,39]. RNA silencing mediates *N. tabacum* resistance [40,41] and tolerance [25,42-44] to the nepoviruses *Tomato black ring virus* and *Tobacco ringspot virus*. Although RNA silencing- and SA-based mechanisms of nepovirus resistance have been described, no nepovirus resistance genes have been identified including in well-studied *Vitis* spp. [45], and the diversity and heritability of nepovirus resistance responses are unknown.

Although experimental work has shed light on the effect of allopolyploidy on pest resistance [4,5], very little is currently known about how allopolyploidy could impact evolution of plant virus resistance. The objective of this research was to investigate the relationship between allopolyploidy, geographical origin and genomic bases of basal antiviral responses in *Nicotiana*. The *Nicotiana-*nepovirus pathosystem is a logical choice to test basal (nonspecific) antiviral responses because *Nicotiana* species are generally inbreeding [10], nepovirus strains are genetically stable [46] and these plants and viruses have not coevolved. In this study, we tested the nepovirus resistance status of *Nicotiana* and ascertained heritability using synthetic allopolyploids. We also tested whether the resistance is local or systemically acquired. The central hypotheses were that greater or lesser basal resistance could be explained by geography and ancestry, and that allopolyploids exhibit greater levels of virus resistance than diploids.

## Results

### Test for virus presence

Twenty-four *Nicotiana* species and synthetic allopolyploids of distinct geographic origins were evaluated for their reaction to infection with GFLV strains GHu and F13, and ToRSV strain AP (Table 1). Since GFLV-GHu displays levels of virulence intermediate to that of GFLV-F13 and ToRSV-AP in most *Nicotiana* species, plants were primarily assessed for resistance to GFLV-GHu. Each plant-virus combination was sampled at three or more time points except when a definite resistance or susceptibility determination could be made in the first or second apical leaf i.e. for GFLV-F13-inoculated 4 × (*N. sylvestris* × *N. tomentosiformis*), 4 × (*N. glutinosa* × *N. tabacum*), 4 × (*N. sylvestris* × *N.otophora*) and 4 × (*N. rustica* × *N. tabacum*) (sampled once), and GFLV-GHu-inoculated 4 × (*N. glutinosa* × *N. tabacum*) and *N. goodspeedii* (sampled twice). All panels were surveyed for virus presence in every plant [populations of four to 32 (median 17) plants], except for GFLV-F13-inoculated 2 × (*N. tabacum* × *N. benthamiana*), where 23 plants in an original population of 70 plants was sampled for virus presence in apical leaves in a stratified sampling approach.

**Table 1:** **Sources of *Nicotiana *** **species and synthetic allopolyploids used in this study**

***Nicotiana *** **sp.**	**Authority**	**Accession**^**a**^	**Germplasm source**	**PI number**	**Origin**^**b**^	**Provider**^**c**^
*attenuata*	Torr. ex S. Watson	N/A	Bureau of Land Management	W6 27220	SW US Pinyon forest	NCSU
*goodspeedii*	H.-M. Wheeler	25-G	USDA ARS Beltsville	NSL 8663	Australia	NCSU
*obtusifolia*	M. Martens and Galeotti	TW98	USDA ARS Beltsville	555543	SW US/ NW Mex	NCSU
*debneyi*	Domin.	TW36	N/A	N/A	Australia	NCSU
*kawakamii*	Y. Ohashi	TW72	Iwata Tobacco Experiment Station	459106	Bolivia	NCSU
*otophora*	Griseb.	TW97	Servicio Agricola Inter-Americano	302477	Ibanex Province, Bolivia	NCSU
*paniculata*	L.	TW100	C. Rick, Univ Calif	241769	Peru	NCSU
*setchelii*	Goodsp.	TW121	USDA ARS Beltsville	555557	Peru	NCSU
*suaveolens*	Lehm.	TW128	CSIRO	230960	Australia	NCSU
*tomentosiformis*	Goodsp.	TW142	USDA ARS Beltsville	555572	Bolivia	NCSU
*glauca*	Graham	N/A	World Seed Supply, Mastic Beach, NY	N/A	Bolivia or Argentina	commercial source
*glutinosa*	L.	N/A	World Seed Supply, Mastic Beach, NY	N/A	Bolivia, Ecuador, Peru	commercial source
*rustica*	L.	N/A	World Seed Supply, Mastic Beach, NY	N/A	Bolivia, Ecuador or Peru	commercial source
*sylvestris*	Speg. and Comes	N/A	Botanical Interests, Inc.; Broomfield, CO	N/A	Bolivia or Argentina	commercial source
*benthamiana*	Domin.	N/A	N/A	N/A	Australia	R. Provvidenti; Cornell
*clevelandii*	A. Gray	N/A	N/A	N/A	SW US or NW Mexico	R. Provvidenti; Cornell
*tabacum* cv. Xanthi	L.	N/A	N/A	N/A	Domesticated	R. Provvidenti; Cornell
**Hybrid maternal parent**	**Hybrid paternal parent**	**PI number**	**Hybrid accession reference**	**ploidy**	**Creator**	**Donor**
*sylvestris*	*tomentosiformis*	555722	TH37	amphidiploid	L. Burk; Prosser, WA	NCSU
*rustica* var. *brasilia*	*tabacum* cv. Boltons special	555701	TH34	amphidiploid	Anon.	NCSU
*sylvestris*	*otophora*	555721	TH32	amphidiploid	L. Burk; Prosser, WA	NCSU
*quadrivalvis*	*tabacum* cv. Red Russian	555515	TH1	amphidiploid	USDA ARS Beltsville	NCSU
*glutinosa*	*tabacum* cv. Red Russian	555520	TH10	amphidiploid	USDA ARS Beltsville	NCSU
*debneyi*	*clevelandii*	555699	TH15	amphidiploid	Cameron, UC Berkeley	NCSU
*tabacum* cv. Turkish Sam. (*nn*) S9-7	*benthamiana*	N/A	hybrid 230	amphihaploid	G.B. Collins; Lexington, KY	Kentucky State U.

^a^N/A: not available.

^b^Specific origin of the accession is given, where known. Otherwise, the endemic range of the species according to [10] is listed.

^c^NCSU: North Carolina State University.

DAS-ELISA was used to determine virus presence or absence for 2719 GFLV samples and 536 ToRSV samples in 48 plant-virus combinations. DAS-ELISA absorbance values had a bimodal distribution, which allowed a clear delineation of virus-positive from virus-negative samples. Infection frequencies at each leaf position in each virus-host sample group were summed to calculate virus incidence, which was the dependent variable in correlation analyses. The trajectory of virus incidence among leaf positions in given host-virus combinations were evaluated to generate six discrete resistance categories. Based on the spatial distribution of virus in host plants, host-virus interactions were labeled ‘susceptible’, ‘immune’ or one of four categories of recovery (‘early’, ‘intermediate’ and late’), or ‘delayed susceptibility’.

### Symptoms

Virus-inoculated plants were checked regularly for symptoms. The only instances of visible symptoms were for GFLV-GHu on *N. benthamiana, N. clevelandii, N. goodspeedii* and 2 × (*N. tabacum* × *N. benthamiana*), and for ToRSV-AP on *N. benthamiana* and 2 × (*N. tabacum* × *N. benthamiana*). GFLV-GHu symptoms on *N. benthamiana* and *N. clevelandii* were consistent with those previously described [47], and included vein clearing on *N. benthamiana* and amorphous ring-like mottling on *N. clevelandii*. GFLV-GHu symptoms on *N. goodspeedii* included vein clearing analogous to that observed for *N. benthamiana*. GFLV-GHu symptoms on the 2 × (*N. tabacum* × *N. benthamiana*) were composed of non-necrotic ringspots on the first or second leaf position. ToRSV-AP symptoms on *N. benthamiana* were similar to those previously described [48], and included stunting, severe mottling, and necrosis from which the plant ultimately recovered. ToRSV-AP caused mild mottling and slight stunting on 2 × (*N. tabacum* × *N. benthamiana*) and symptoms were not observed on *N. tabacum* cv. Xanthi.

### Inoculated leaf infection

**Figure 1:**
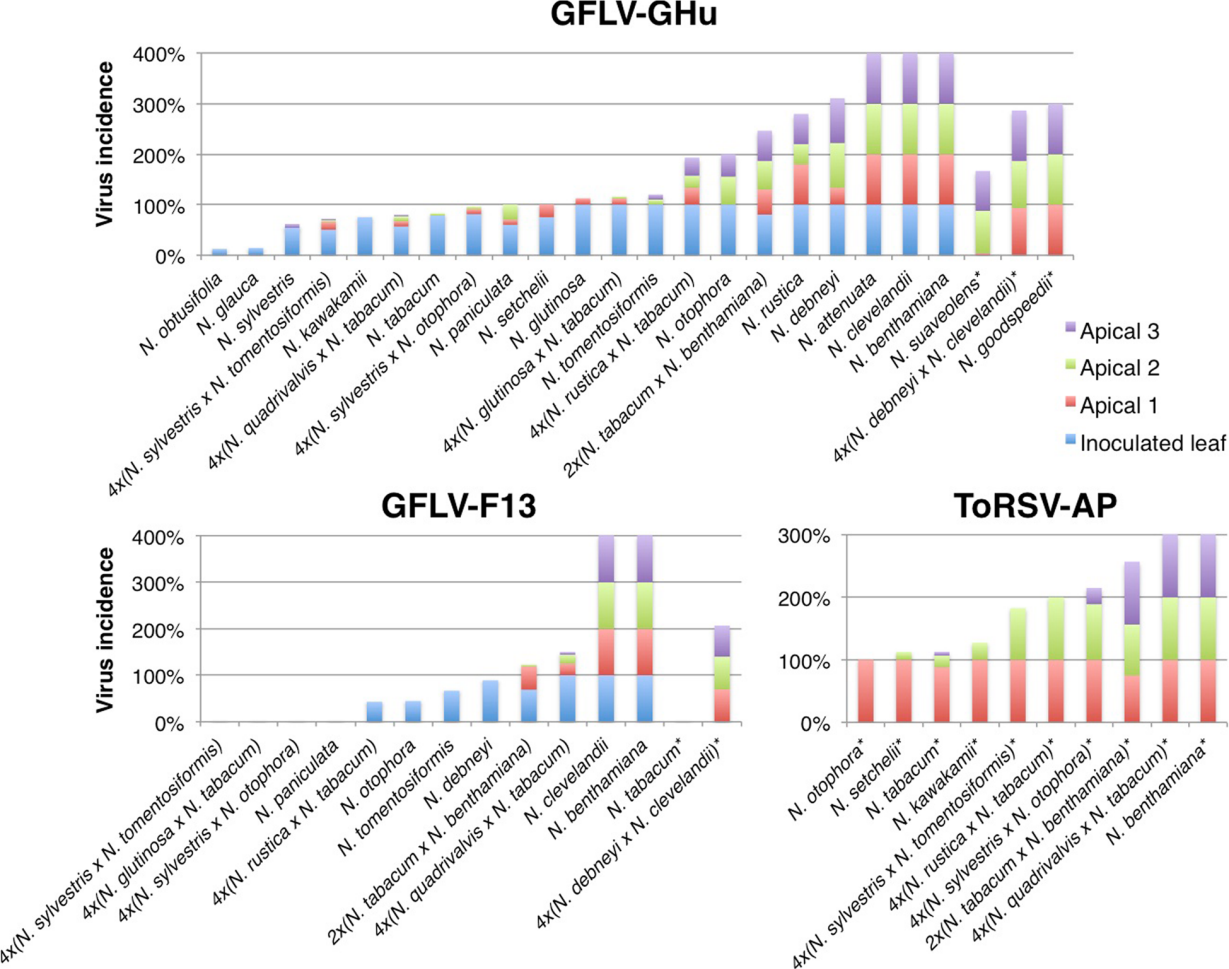
**Infection frequencies in inoculated and apical leaves of populations of plants tested for resistance to *Grapevine fanleaf virus *(GFLV) strains GHu and F13, and *Tomato ringspot virus *(ToRSV) strain AP.** The percent virus incidence is indicated for inoculated and apical leaves. Asterisks (*) after plant names indicate that the inoculated leaves in the plant-virus combination were not tested.

DAS-ELISA revealed different frequencies of virus infection in inoculated leaves (Figure 1). Some host-virus combinations consistently produced absorbance values below the virus detection threshold, which reflects immunity or perhaps limited subliminal (single cell) infections. 4 × (*N. sylvestris* × *N. tomentosiformis*), 4 × (*N. sylvestris* × *N. otophora*), 4 × (*N. glutinosa* × *N. tabacum*) and *N. paniculata* exhibited immunity to GFLV-F13 in inoculated leaves. Some host-virus combinations resulted in less than 50% inoculated leaf infection including GFLV-GHu-inoculated *N. obtusifolia* (13%) and *N. glauca* (14%), and GFLV-F13-inoculated 4 × (*N. rustica* × *N. tabacum*) (43%) and 4 × (*N. glutinosa × N. tabacum*) (44%) (Additional file 1: Table S1). All other tested host-virus combinations produced 50% or greater inoculated leaf infection (Additional file 1: Table S1). Since GFLV-GHu always produced infections in inoculated or apical leaves, and ToRSV-AP inoculations always produced some frequency of infection in the first apical leaf, there is no immunity within this *Nicotiana* panel to these two viruses (Table 2).

**Table 2:** ***Grapevine fanleaf virus *** **and *Tomato ringspot virus *** **resistance ratings of *Nicotiana *** **species and synthetic allopolyploids**

**Section**^**a**^	**Origin**	***x*** **=**	***Nicotiana *** **species**	**GFLV-GHu**^**b**^	**GFLV-F13**^**c**^	**ToRSV-AP**
*Tomentosae*	Bolivia	12	*N. otophora*	5	2	3
	Bolivia	12	*N. tomentosiformis*	2	2	
	Bolivia	12	*N. kawakamii*	2		3
	Peru	12	*N. setchelii*	3		3
*Paniculatae*	Peru	12	*N. paniculata*	3	1	
*Noctiflorae*	Bolivia	12	*N. glauca*	2		
*Sylvestres*	Bolivia	12	*N. sylvestris*	2		
*Undulatae*	Peru	12	*N. glutinosa*	3		
*Trigonophyllae*	SW US	12	*N. obtusifolia*	2		
*Petunioides*	SW US	12	*N. attenuata*	6		
*Suaveolentes*	Australia	16	*N. suaveolens*	5		
	Australia	19	*N. benthamiana*	6	6	6
	Australia	20	*N. goodspeedii*	6		
	Australia	24	*N. debneyi*	4	2	
*Polydicliae*	SW US	24	*N. clevelandii*	6	6	
*Rusticae*	Bolivia	24	*N. rustica*	4		
*Nicotiana*	Domesticated	24	*N. tabacum*	2	1 or 2	3
Wide crosses (synthetic allopolyploids)		24	4*x*(*N. sylvestris* x *N. otophora*)	3	1	3
		24	4*x*(*N. sylvestris* x *N. tomentosiformis*)	3	1	3
		36	4*x*(*N. glutinosa* x *N. tabacum*)	3	1	
		43	2*x*(*N. tabacum* x *N. benthamiana*)	4	3	5
		48	4*x*(*N. quadrivalvis* x *N. tabacum*)	3	3	6
		48	4*x*(*N. rustica* x *N. tabacum*)	4	2	3
		48	4*x*(*N. debneyi* x *N. clevelandii*)	5	4	

^a^Species and synthetic allopolyploids are referenced by their sections within the genus *Nicotiana*, primary location of origin, and their haploid chromosome numbers according to [10].

^b^Categories of resistance (1, most resistant, through 6, most susceptible) are indicated for each virus-host combination tested.

^c^Boxes without resistance ratings represent virus-host combination not tested.

### High resistance interactions

Virus-host combinations yielding no detectable virus in inoculated leaves (and apical leaves) were designated as immune (category 1). Immunity was observed for *N. paniculata,* 4 × (*N. sylvestris* × *N. tomentosiformis*)*,* 4 × (*N. sylvestris* × *N. otophora*) and 4 × (*N. glutinosa* × *N. tabacum*) inoculated with GFLV-F13 (Figure 1; Table 2)*.* GFLV-F13-inoculated *N. tabacum* did not produce apical leaf infection, but whether this plant is immune (category 1) or possesses early recovery (category 2) to GFLV-F13 is uncertain because inoculated leaves were not tested. All tested members of section *Tomentosae*, *N. debneyi* and 4 × (*N. rustica* × *N. tabacum*) exhibited early recovery (category 2) to GFLV-F13 (Table 2). *N. obtusifolia*, *N. glauca*, *N. sylvestris*, *N. kawakamii*, *N. tabacum* and *N. tomentosiformis* exhibited early recovery after GFLV-GHu inoculation. Early recovery was not observed for these species in response to inoculation with ToRSV-AP (Table 2).

### Moderate resistance interactions

Late recovery (category 3) was the most frequent host-virus interaction phenomenon observed in this test panel, and was seen for all virus isolates tested. All tested members of section *Tomentosae*, 4*×*(*N. rustica* × *N. tabacum*)*, N. tabacum* and resynthesized allopolyploids involving possible *N. tabacum* progenitor species [4 × (*N. sylvestris* × *N. tomentosiformis*) and 4 × (*N. sylvestris* × *N. otophora*)] showed late recovery to ToRSV-AP (Table 2)*.* 2 × (*N. tabacum* × *N. benthamiana*) and 4 × (*N. quadrivalvis* × *N. tabacum*) showed late recovery to GFLV-F13, and 4*x*(*N. quadrivalvis* × *N. tabacum*), 4 × (*N. sylvestris* × *N. otophora*)*,* 4 × (*N. sylvestris* × *N. tomentosiformis*)*,* 4 × (*N. glutinosa* x *N. tabacum*), *N. glutinosa, N. paniculata* and *N. setchelii* showed late recovery to GFLV-GHu (Table 2)*.* Intermediate recovery (category 4), characterized by fluctuation of virus incidence over three or more leaf axes (typically between 33% and 67%, Additional file 1: Table S1), was observed in GFLV-GHu-inoculated *N. debneyi, N. rustica,* 4 × (*N. rustica* × *N. tabacum*) and 2 × (*N. tabacum × N. benthamiana*), and in GFLV-F13-inoculated 4 × (*N. debneyi* × *N. clevelandii*) (Table 2).

### Low or no resistance interactions

Delayed susceptibility (category 5) was observed only in response to GFLV-GHu inoculation of *N. otophora*, *N. suaveolens,* and 4 × (*N. debneyi* × *N. clevelandii*) (Figure 1; Table 2). Plants were designated as susceptible (category 6) when 100% of the plants became infected and virus was present in all tested leaves. *N. benthamiana* and *N. clevelandii* were susceptible to GFLV-F13 and GFLV-GHu, as expected [47], *N. goodspeedii* and *N. attenuata* were susceptible to GFLV-GHu, and *N. benthamiana* and 4 × (*N. quadrivalvis* × *N. tabacum*) were susceptible to ToRSV-AP (Figure 1; Table 2).

### Additive resistance phenomena in synthetic polyploid plants

**Figure 2:**
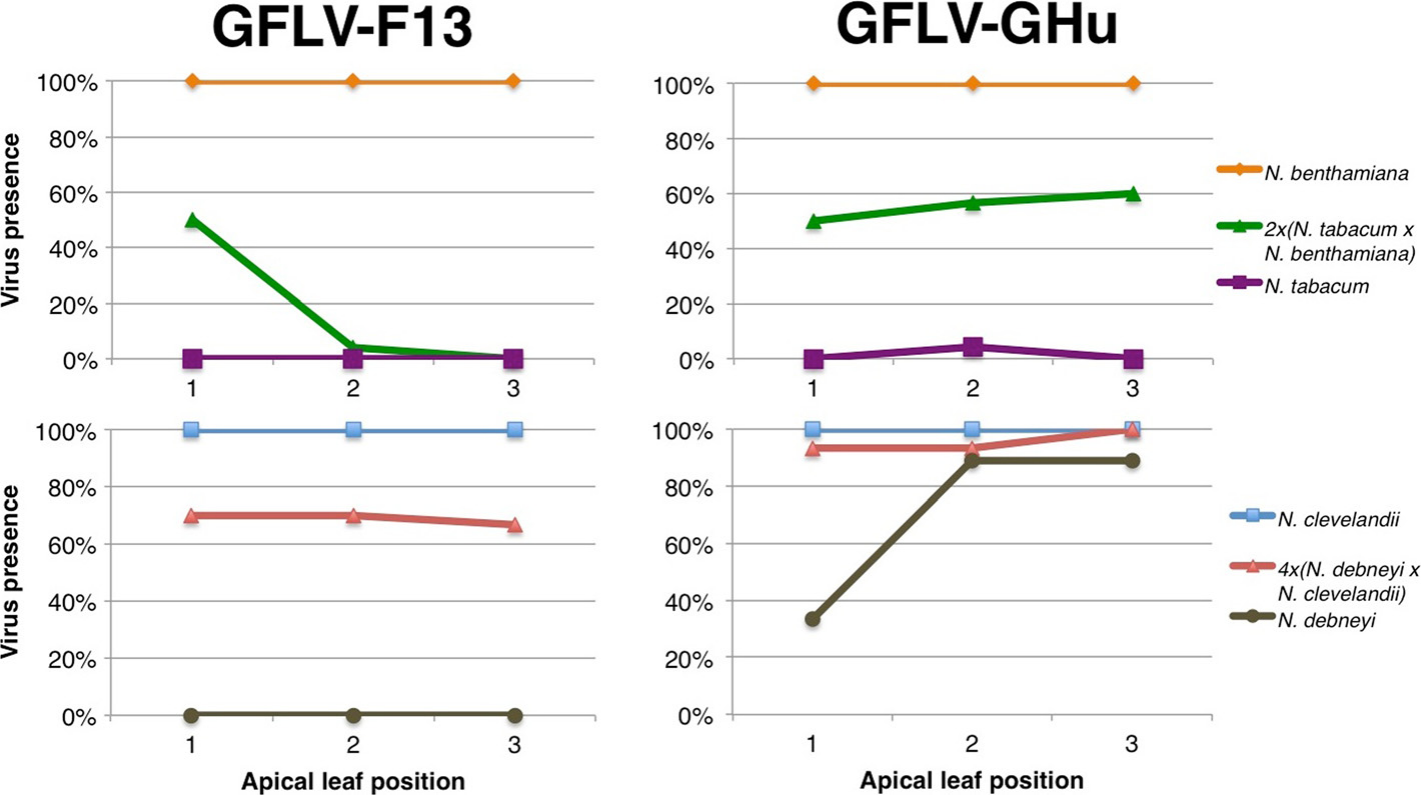
**Effect of synthetic *Nicotiana *allopolyploids on resistance to *Grapevine fanleaf virus *(GFLV) strains F13 (left panels) and GHu (right panels).** *N. tabacum*, *N. benthamiana* and the 2*x*(*N. tabacum* x *N. benthamiana*) amphihaploid (upper panels); and *N. debneyi*, *N. clevelandii* and 4*x*(*N. debneyi* x *N. clevelandii*) allopolyploid (lower panels) were tested for additive resistance.

**Figure 3:**
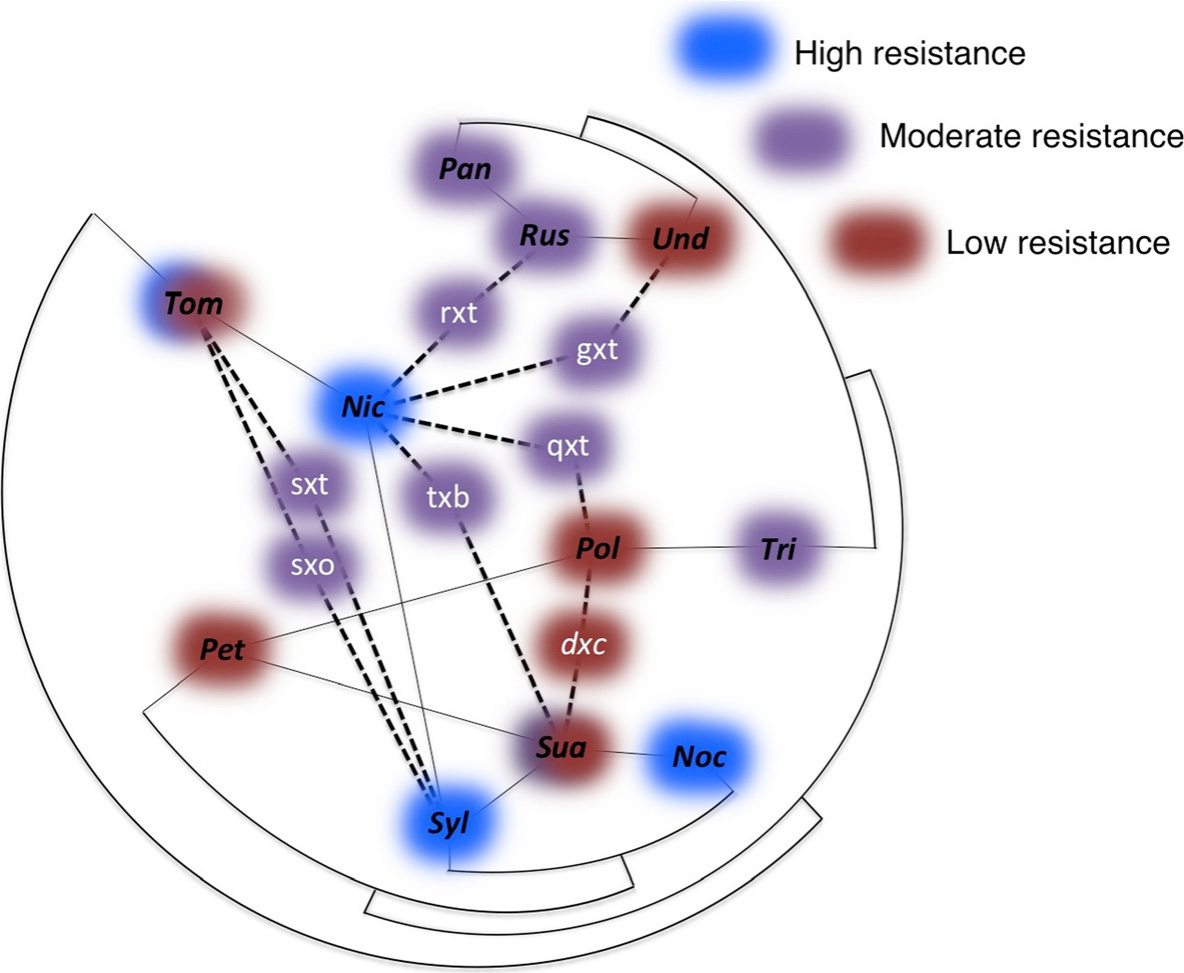
***Grapevine fanleaf virus *strain GHu (GFLV-GHu) resistance categories superimposed on a *Nicotiana *phylogenetic tree modified from Clarkson *et al.***[18]**(curved lines), containing sections (abbreviated in black lettering) with allopolyploid ancestries as established by Clarkson *et al.***[13]**and Kelly *et al.***[14]**(solid straight lines).** Shading surrounding sections denote the resistance category of representative species tested for GFLV-GHu resistance: blue (category 2, early recovery), purple (categories 3 and 4, late or intermediate recovery), or red (categories 5 and 6, delayed or full susceptibility). Representative *Nicotiana* species (sections) used in this study are *N. paniculata* (*Paniculatae, ‘Pan’*), *N. rustica* (*Rusticae, ‘Rus’*), *N. obtusifolia* (*Trigonophyllae, ‘Tri’*), *N. benthamiana, N. debneyi, N. suaveolens* and *N. goodspeedii* (*Suaveolentes, ‘Sua’*), *N. clevelandii* (*Polydicliae, ‘Pol’*), *N. glauca* (*Noctiflorae, ‘Noc’*), *N. sylvestris* (*Sylvestres ‘Syl’*), *N. tabacum* (*Nicotiana ‘Nic’*), *N. glutinosa* (*Undulatae, ‘Und’*) *N. attenuata* (*Petunioides, ‘Pet’*), (*Tomentosae, ‘Tom’*) including *N. kawakamii, N. otophora, N. setchelii* and *N. tomentosiformis*. Members of *Tomentosae* and *Suaveolentes* exhibited different GFLV-GHu resistance profiles and are accordingly dually or triply colored. Descent of synthetic allopolyploids used in this study (white letters) is indicated by dashed lines: *4*x(*N. sylvestris* x *N. tomentosiformis*) (‘sxt’), *4*x(*N. rustica* x *N. tabacum*) (‘rxt’), *4*x(*N. glutinosa* x *N. tabacum*) (‘gxt’), *2*x(*N. tabacum* x *N. benthamiana*) (‘txb’), *4*x(*N. quadrivalvis* x *N. tabacum*) (‘qxt’) and *4*x(*N. debneyi* x *N. clevelandii)* (‘dxc’).

Incompletely dominant virus resistance was observed in synthetic *Nicotiana* allopolyploids. Whereas *N. tabacum* showed high resistance to GFLV-GHu, ToRSV-AP and GFLV-F13, and *N. benthamiana* was fully susceptible to all three virus strains, 2 × (*N. tabacum* × *N. benthamiana*) exhibited delayed susceptibility to GFLV-GHu, intermediate recovery to ToRSV-AP, and late recovery to GFLV-F13 (Figure 2; Table 2). Whereas *N. clevelandii* was fully susceptible to all viruses tested, and *N. debneyi* exhibited early recovery to GFLV-F13 and intermediate recovery to GFLV-GHu, 4 × (*N. debneyi* × *N. clevelandii*) exhibited intermediate recovery to GFLV-F13 and delayed susceptibility to GFLV-GHu (Figure 2, Table 2). The 4 × (*N. rustica* × *N. tabacum*) response to GFLV-GHu was not categorically different than the response of *N. rustica* (both category 4), but the synthetic allopolyploid showed consistently lower incidence of infection in apical leaves (23-40%) compared to *N. rustica* (40-80%), which could reflect the contribution of *N. tabacum* (category 2) to resistance (Figure 1; Additional file 1: Table S1). The intermediate virus resistance observed across *Nicotiana* lineages (Figure 3) suggests quantitative resistance is not due to a single gene with dosage effects, but due to multiple genes with dosage effects.

### Resistance profiles of allopolyploids and their progenitors

We tested two natural allopolyploids (*N. clevelandii* and *N. tabacum*) and the closest relatives of their known progenitors for GFLV-GHu resistance. The closest extant diploid progenitors of *N. clevelandii* are *N. obtusifolia* (maternal genome donor) and *N. attenuata* (paternal genome donor) [13]. While *N. obtusifolia* exhibited an early recovery phenotype (category 2), both *N. clevelandii* and *N. attenuata* showed complete susceptibility (Figure 1; Table 2). *N. tabacum*, its representative maternal genome donor (*N. sylvestris*), and one possible representative paternal genome donor (*N. tomentosiformis*) each exhibited early recovery (category 2). *N. otophora*, another representative of *N. tabacum’s* possible paternal genome donors exhibited delayed susceptibility (category 5) to GFLV-GHu. Resynthesized allopolyploids corresponding to either *N. tabacum* ancestry scenario exhibited late recovery phenotypes (category 3) to GFLV-GHu with low virus incidence levels (Figure 1; Additional file 1: Table S1). Thus, *N. tabacum* exhibits an early recovery phenotype similar to that of its maternal genome donor and of *N. tomentosiformis*, but less resistance than that of *N. otophora* or representative resynthesized allopolyploids. Additionally, species of section *Suaveolentes* showed low or occasionally moderate resistance to GFLV-GHu, while its most closely related proposed paternal genome donor (*N. sylvestris*) [14] showed high resistance (early recovery) (Table 2). While neoallopolyploids showed intermediate GFLV-GHu resistance characteristics, extant allopolyploids did not show intermediate GFLV-GHu resistance characteristics (Figure 3).

### Systemic recovery

Systemic recovery was tested in apical leaves of GFLV-resistant (categories 1 or 3) synthetic allopolyploids 4 × (*N. sylvestris* × *N. tomentosiformis*)*,* 4 × (*N. glutinosa* × *N. tabacum*)*,* and 4 × (*N. sylvestris* × *N. otophora*) (Table 3). Resistance was induced with GFLV-GHu or GFLV-F13, and one upper, apical leaf of each recovered plant was re-inoculated with GFLV-GHu and tested for virus presence. Notably, plants that showed inoculated leaf susceptibility to GFLV-GHu lost this susceptibility in the apical leaf of the recovered plant, no matter whether the resistance was induced with GFLV-F13 or GFLV-GHu (Table 3). GFLV-GHu was occasionally detected in the apical inoculated leaf of GFLV-GHu-recovered plants encompassing two of 21 plants in 4 × (*N. sylvestris* × *N. tomentosiformis*) and one of nine plants in 4 × (*N. sylvestris* × *N. otophora*). Of the plants that did not acquire systemic recovery, the possibility of the originally-inoculated GFLV-GHu infected these apical leaves cannot be excluded given that late recovery does not bar the virus from infecting the fourth leaf position, albeit at a low incidence.

**Table 3:** **Systemic recovery from *Grapevine fanleaf virus *** **(GFLV) strains F13 and GHu**

		**1st inoculation**^**a**^	**Resistance response**	**Systemic recovery**^**b**^
4*x*(*N. sylvestris* x *N. tomentosiformis*)		GFLV-GHu	Late recovery	90%
		GFLV-F13	Immunity	100%
4*x*(*N. glutinosa* x *N. tabacum*)		GFLV-GHu	Late Recovery	100%
		GFLV-F13	Immunity	100%
4*x*(*N. sylvestris* x *N. otophora*)		GFLV-GHu	Late Recovery	89%

^a^GFLV-GHu was inoculated to the fourth apical leaf following induction of resistance (resistance response against the virus in the 1^st^ inoculation is indicated).

^b^Plants (n = 9 to 29) were characterized as having systemic recovery if GFLV was undetectable at five days post-inoculation.

### Relationship between host geographic origin and virus resistance

Australian and North American accessions generally displayed greater levels of susceptibility than South American accessions to all virus strains tested (Table 2). The Australian species *N. benthamiana* and the North American species *N. clevelandii* were fully susceptible to all viruses tested, and Australian species *N. debneyi, N. suaveolens* and *N. goodspeedii*, and North American species *N. attenuata* and *N. quadrivalvis* displayed lower levels of resistance than South American species to GFLV-GHu individually or in hybrid backgrounds (Table 2). Exceptions to these geography-based resistance trends included the *N. debneyi* (Australia) early recovery response to GFLV-F13, the *N. obtusifolia* (North America) early recovery response to GFLV-GHu, and the *N. otophora* (South America) delayed susceptibility response to GFLV-GHu. Overall, origin had a significant (*P* < 0.0001) and moderate correlation for GFLV-GHu incidence when hybrids were excluded from the analysis (*r* = 0.683) and a weaker correlation (*r* = 0.5422, *P* < 0.0001) when hybrids were included, with South American species showing greater resistance than Australian species, which in turn showed greater resistance than species from the Southwest US. Because the effect of section cannot be separated from the effect of origin (Table 2), the effect of origin on virus resistance could reflect phylogenetic factors.

### Limited relationship between host ploidy level and virus resistance

There was a weak association between ploidy level and virus susceptibility. For example, *n* = 12 diploids from section *Tomentosae* generally displayed greater levels of resistance than *n* = 16-24 allopolyploids of section *Suaveolentes,* and similar levels of resistance to *N. tabacum* and *N. rustica* (*n* = 24) (Table 2). The correlation between GFLV-GHu incidence and chromosome number was low (*r* = -0.036) and nonsignificant (*P* = 0.2597) when hybrids were included in the analysis, and low (*r* = -0.286) but significant (*P* < 0.0001) when hybrids were excluded, indicating that increasing ploidy is weakly negatively related to GFLV-GHu virus incidence among extant *Nicotiana* species. These results indicate that increasing ploidy is correlated with slightly greater virus susceptibility, but that the trend is abolished when synthetic allopolyploids are taken into account.

### Other trends in virus resistance

Members of section *Tomentosae* produced higher inoculated leaf infection rates (75-100%) for GFLV-GHu than for GFLV-F13 (44-67%) (Additional file 1: Table S1). Every tested member of section *Tomentosae* produced an early recovery phenotype for GFLV-F13 and a late recovery phenotype for ToRSV-AP (Table 2). Members of section *Tomentosae* showed variability in response to GFLV-GHu, where *N. kawakamii* and *N. tomentosiformis* exhibited early recovery, *N. setchelii* displayed late recovery, and *N. otophora* showed delayed susceptibility (Table 2). The delayed susceptibility of *N. otophora* to GFLV-GHu was masked in the 4 × (*N. sylvestris* × *N. otophora*) synthetic allopolyploid, which reflected the early recovery of *N. sylvestris* to GFLV-GHu (Table 2). Early recovery was also observed for *N. tabacum* inoculated with GFLV-GHu*,* a species believed to have evolved from a *N. sylvestris* × *N. otophora* or *N. sylvestris* × *N. tomentosiformis* hybridization event [10]. Members of section *Suaveolentes* exhibited intermediate or low resistance to the nepovirus strains tested, except for *N. debneyi*, which displayed early recovery after inoculation with GFLV-F13 (category 2) (Table 2).

*N. tabacum* and its corresponding resynthesized allopolyploids [4 × (*N. sylvestris* × *N. otophora*) and 4 × (*N. sylvestris* × *N. tomentosiformis*)] exhibited high or moderate virus resistance phenotypes for each virus tested (Table 2). Both resynthesized allopolyploids are immune to GFLV-F13, and *N. tabacum* also displays high resistance to this virus. *N. tabacum* and its resynthesized allopolyploids showed late recovery to ToRSV-AP, though *N. tabacum* frequently had lower frequencies of infection at any given leaf position than its corresponding neoallopolyploids (Figure 1; Additional file 1: Table S1). The response of *N. tabacum* and the synthetic allopolyploids 4 × (*N. sylvestris* × *N. otophora*) and 4 × (*N. sylvestris* × *N. tomentosiformis*) to GFLV-GHu were similar in terms of inoculated leaf infection, but *N. tabacum* showed early recovery whereas the neoallopolyploids showed late recovery, though the overall apical virus incidence levels were similar (Additional file 1: Table S1). The recovery responses of *N. tabacum* to GFLV and ToRSV inoculation confirm previous reports [38,39].

Synthetic polyploids formed from resistant and susceptible species frequently displayed resistance in the moderate categories (Figure 3). 2 × (*N. tabacum* × *N. benthamiana*) and 4 × (*N. debneyi* × *N. clevelandii*) exhibited intermediate resistance phenotypes after inoculation with GFLV-GHu and GFLV-F13 compared to their parents (Figure 2; Table 2). The same was true for the 2 × (*N. benthamiana* × *N. tabacum*) response to ToRSV-AP (Table 2). An intermediate level of apical leaf infection was also seen in the 4 × (*N. rustica × N. tabacum*) response to GFLV-GHu (Figure 1).

ToRSV-AP typically produced equal or greater categorical ratings than GFLV-GHu, and GFLV-GHu always produced equal or higher category ratings than GFLV-F13 (Table 2). An exception to this virulence trend was that *N. otophora* and 4 × (*N. rustica × N. tabacum*) showed lower resistance (higher category ratings) to GFLV-GHu than to ToRSV-AP (Table 2). Virulence differences between GFLV-F13 and GFLV-GHu were highly apparent in synthetic allopolyploid plants with resistant and susceptible parents, including 2 × (*N. tabacum* × *N. benthamiana*), 4 × (*N. rustica × N. tabacum*), and 4 × (*N. debneyi* x *N. clevelandii*) (Table 2; Figure 2)*.* There was a significant (*P* < 0.0001) but weak (*r* = 0.406) correlation between virus composition and infection frequencies across plant genotypes (species or synthetic allopolyploids).

According to individual components of *χ*^2^ in the contingency table that compared observed and expected virus incidence frequencies for each virus at each leaf position, there is a higher virus incidence in the first apical leaf than expected for ToRSV-AP; conversely, there is less virus incidence in the first apical leaf than expected for GFLV-F13 (data not shown). Expected and observed apical virus incidence values are similar for GFLV-GHu. These results suggest that ToRSV-AP displays higher virulence and GFLV-F13 displays lower virulence than GFLV-GHu in this panel of *Nicotiana* species.

## Discussion

A spectrum of plant resistance and viral virulence was observed in the present *Nicotiana*-nepovirus panel. Changes in virus incidence were characterized using DAS-ELISA on multiple leaves of large samples of plants (Figure 1; Additional file 1: Table S1) and used to distill six categories of host resistance (immunity, susceptibility, and four categories of recovery) from which *Nicotiana* species, synthetic allopolyploids and viruses were compared. While all host-virus combinations exhibiting low leaf inoculation frequencies (<50%) exhibited early recovery, this phenotype was frequently associated with a high infection frequency (>50%) in inoculated leaves (Figure 1). Moderate or high leaf inoculation frequencies (≥50%) were associated with an entire range of resistance and susceptibility phenotypes (category 2 through category 6) (Figure 1). Within individual plant genotypes, ToRSV-AP generally produced higher susceptibility ratings than GFLV-GHu, and GFLV-GHu always produced an equal or greater susceptibility rating than GFLV-F13 (Table 2), and the correlation between virus identity and virus incidence ratings were significant. The spectra of quantitative resistance displayed by *Nicotiana* accessions and virulence among nepoviruses suggest the role of multiple interacting alleles from *Nicotiana* accessions and nepoviruses in the determination of the ultimate infection outcomes. Similar plant genotype by virus genotype interactions were observed in a panel of 21 *Arabidopsis* accessions challenged with three *Cucumber mosaic virus* isolates [49].

The full susceptibility seen for 4 × (*N. quadrivalvis* × *N. tabacum*) and delayed susceptibility of the 2 × (*N. tabacum* × *N. benthamiana*) responses to ToRSV raises the interesting possibility that *N. quadrivalvis* and *N. benthamiana* may possess a dominant ToRSV susceptibility factor in *N. tabacum* backgrounds. The observation of ringspot symptoms on the GFLV-GHu-inoculated 2 × (*N. tabacum* x *N. benthamiana*) amphihaploid suggests that the vein clearing symptomology typical of *N. benthamiana* infection [47] is a recessive trait. Similarly, while ToRSV-AP produced necrosis on *N. benthamiana*, necrosis was not observed on the 2 × (*N. tabacum × N. benthamiana*) amphihaploid or on *N. tabacum*. The absence of *N. tabacum-*ToRSV necrotic ringspot symptoms was unexpected given previous reports [39,50]. The lack of hypersensitive responses observed in this host panel is consistent with the lack of involvement of a specific gene-for-gene recognition system in *Nicotiana-*GFLV and *Nicotiana*-ToRSV interactions. This lack of hypersensitive response and the absence of coevolutionary history between *Nicotiana* and GFLV or ToRSV supports the idea that resistance or susceptibility is due to the interaction of broad-spectrum immune responses and virulence factors [51].

**Figure 4:**
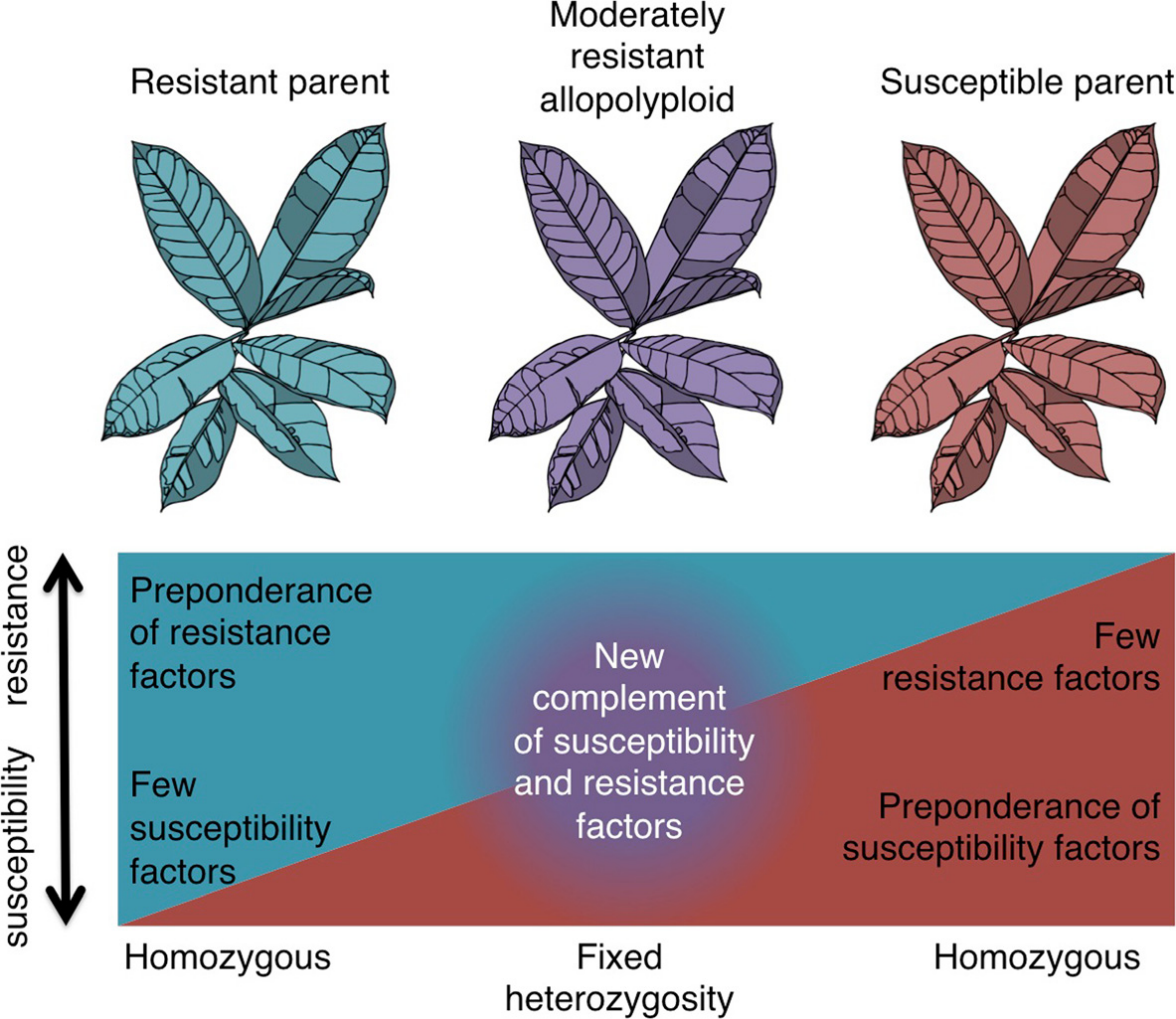
**Pictographic description of the modified matching allele model applied to the additive resistance hypothesis.** Resistant and susceptible parents (e.g. diploid progenitors of an allopolyploid) carry unique complements of resistance factors (blue) and susceptibility factors (red). The allopolyploid plant would maintain a mix of resistance and susceptibility factors from each parent (fixed heterozygosity), and also would be expected to exhibit unique (nonadditive) expression profiles of resistance and susceptibility factors.

Most plants in the host panel used in this study recovered from virus infection after infection was initially established in inoculated leaves. Recovery from virus infection can be controlled by simple or complex host plant genetics, and can be countered by effective pathogen virulence factors [30,52,53]. Host plant and pathogen genotype determined the level of plant recovery to GFLV (Figure 2). Compatibility between host and viral components is a prerequisite for infection in the matching allele model [54,55]. The partial resistance phenotypes observed in this study do not fit with the strict bimodality of the matching allele concept. However, a modified matching allele model that allows for partial compatibility and limited infection [1,55] (Figure 4) could explain the range of resistance and virulence observed in the *Nicotiana*-nepovirus interactions observed here.

The intermediate resistance responses of *Nicotiana* neoallopolyploids are congruent with the additive resistance hypothesis proposed by Fritz *et al.*[56]. By applying the modified matching allele model to the additive resistance hypothesis, we theorize that susceptible parents contribute susceptibility alleles and resistant parents contribute resistance alleles, and their neoallopolyploids contain novel combinations of resistance and susceptibility factors (Figure 4). Neoallopolyploids would possess a greater number of matching alleles than their more resistant parent, but the dosage of resistance factors would be reduced compared to the resistant parent. Furthermore, non-additive gene expression, which is commonly observed in allopolyploids and other hybrids [57-59], could modify expression of resistance and susceptibility alleles (Figure 4).

While the identities of the *Nicotiana*’s nepovirus resistance alleles are unknown, re-inoculation experiments (Table 3) show that the resistance signal is translocated to result in systemic recovery. Because the *N. tabacum* ToRSV resistance response appears to be SA-mediated [39], susceptibility alleles conferred by *N. benthamiana* in the 2 × (*N. tabacum* × *N. benthamiana*) hybrid could allow ToRSV to quantitatively inhibit SA biosynthesis, affect conversion of SA to an alternate derivative, or vitiate downstream SA-activated resistance responses [60,61]. Similarly, null or ineffective RNA silencing alleles present in susceptible backgrounds could conceivably compromise RNA silencing-mediated virus resistance in hybrids [62]. These hypotheses are consistent with Fraser’s model of virus resistance [29], which postulates that the effects of resistance alleles are proportional to their dosage and levels of influence on resistance pathways.

Although animal allopolyploids frequently show dominant parasite susceptibility [4,5,63], *Nicotiana* neoallopolyploids exhibit virus resistance greater than one but not both of their parents (Figure 3). In cases where both parents were either resistant or susceptible, the neoallopolyploid displayed a resistance response similar to their parents, and thus there was no inherent penalty or benefit from hybridization or genome duplication (Figure 3). Contrary to the model that neoallopolyploid plants could face a depression of innate immunity [64], our findings suggest that allopolyploidization itself did not penalize *Nicotiana* for virus resistance.

**Figure 5:**
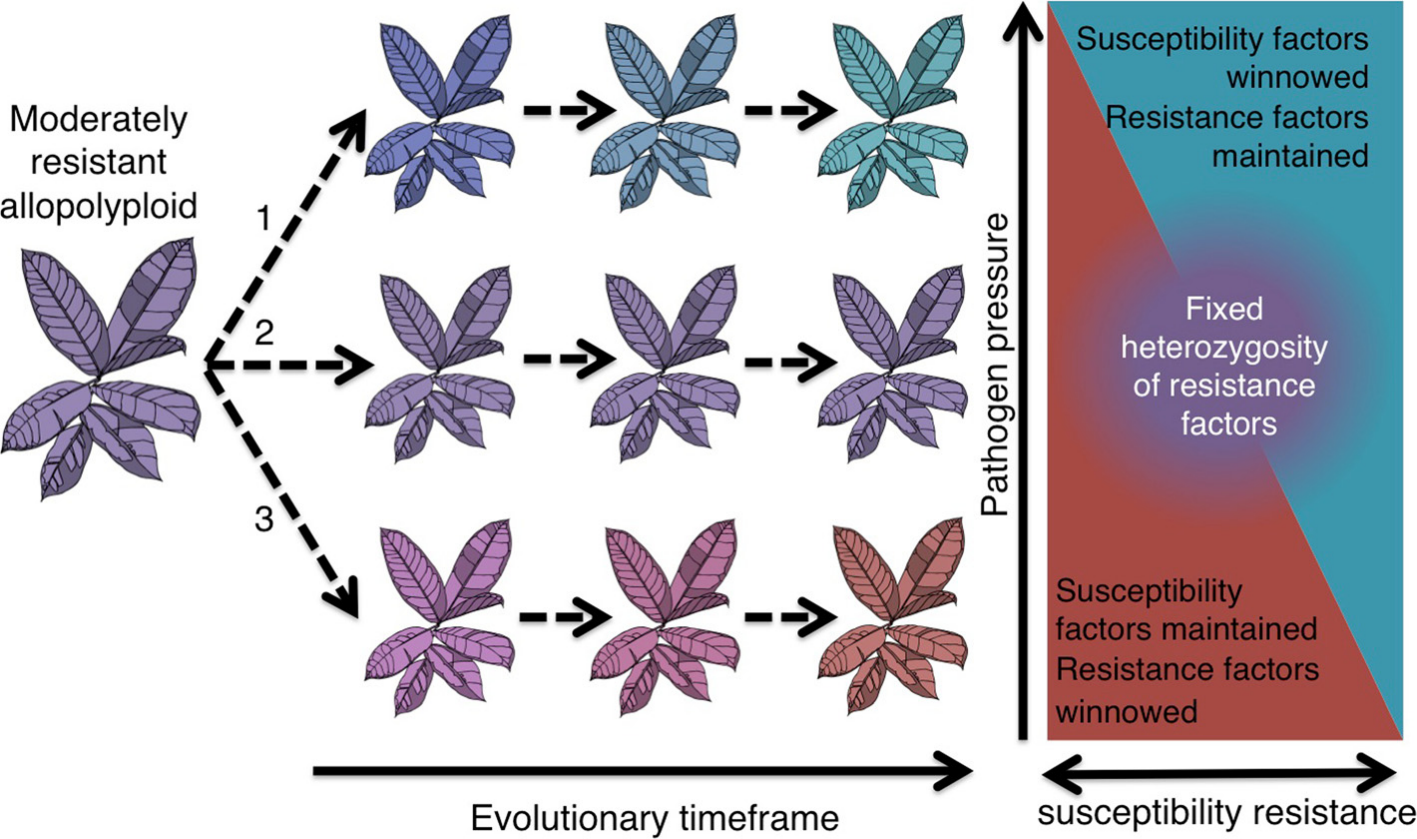
**Model of changes in quantitative innate virus resistance from a moderately resistant progenitor exhibiting fixed heterozygosity for resistance genes (e.g. a neoallopolyploid).** Random divergence of the allopolyploid progeny leads to several possible lineages containing different resistance or susceptibility phenotypes whose existence depends on drift and pathogen pressure. High pathogen pressure would select for the loss of susceptibility factors and maintenance and gain of resistance factors (trajectory 1, top row). Moderate or irregular pathogen pressure would maintain an equilibrium of resistance and susceptibility factors within the plant population (trajectory 2, middle row). Low pathogen pressure would remove the selective advantage of maintaining resistance factors, and could result in the loss of resistance factors and the maintenance of susceptibility factors (trajectory 3, bottom row).

‘Revolutionary changes’ that accompany polyploidy can be distinguished from ‘evolutionary changes,’ which follow allopolyploidization [65,66]. The maintenance of virus resistance in *N. tabacum* contrasts with the apparent loss of virus resistance in section *Polydicliae*, which did not maintain partial virus resistance imparted by its likely maternal genome donor (*N. obtusifolia*) (Figure 3). Similarly, members of *Suaveolentes* exhibited high degrees of virus susceptibility despite the resistance of their paternal genome donor’s closest relative (*N. sylvestris*). Low virus resistance in sections *Polydicliae* and *Suaveolentes* suggests genetic drift and/or selection conferred a loss of virus resistance inherited by neoallopolyploids. *Nicotiana* neoallopolyploids show gene loss and neofunctionalization [12,16,18]. Since favorable alleles have a lower chance of becoming fixed in allopolyploids than diploids [6], drift could have resulted in losses of innate immunity alleles in the *Polydicliae* and *Suaveolentes* lineages (Figure 5).

The correlation of higher levels of virus resistance in South American *Nicotiana* species than North American and Australian species suggest that geographic influences had a major effect on the efficacy of antiviral resistance responses. Alternatively, because plant taxon (section) is frequently inseparable from origin, there is a possibility that phylogeny rather than origin could account for virus resistance. In either scenario, long-term biota-specific interactions would be critical factors to select for improved virus resistance. Existing virus resistance alleles could be maintained or enhanced if virus challengers perennially recur (trajectories 1 or 2), or virus resistance alleles could be lost if virus challenges diminish (trajectory 3) (Figure 5). Comparison of several allopolyploids used in these virus resistance experiments suggest that *N. tabacum*, endemic to the relatively large and competitive biome of the Eastern Andes has followed trajectory 1, while members of sections *Suaveolentes* and *Polydicliae*, endemic to the relatively isolated biota of Australia and Southwest US, respectively, have followed trajectories 2 or 3 (Figure 5). Because *N. debneyi* and *N. benthamiana* are monophyletic [10], but *N. debneyi* shows greater antiviral resistance than *N. benthamiana* (Table 2), random or selection-based processes may have driven divergence of innate immune functions within this allopolyploid lineage. The sister allopolyploids *N. clevelandii* and *N. quadrivalvis* have similarly diverged for herbivory resistance responses [67].

Changes in immune function due to allopolyploidy could precipitate changes in challenging pathogens, and prompt a Red Queen-type evolutionary response between the plant and pathogen [5]. Ineffective innate immune systems could allow otherwise ill adapted viruses to acquire more effective virulence factors and erode quantitative resistance [68,69]. An allopolyploid that can endure colonization by a pathogen or pest and that permits pathogen adaptation to an otherwise resistant host progenitor is referred to as a hybrid bridge [70]. Rather than escaping virus infection, allopolyploids could furnish another niche for viruses to expand their host ranges.

## Conclusion

The Red Queen Hypothesis explains how resistance and virulence temporally change in parasitic relationships. Allopolyploidy might represent an opportunity for plant hosts to break the Red Queen cycle of coevolution by gaining a new complement of dominant resistance factors, but the potential for allopolyploids to experience an epochal gain in innate immune function may be compromised by the inheritance of susceptibility alleles or genetic dysfunctionality caused by hybridization. The *Nicotiana*-nepovirus interaction sheds light on potential dynamics of how allopolyploidy may affect innate immunity. Based on a detailed survey of the interaction of non-coevolved plant and virus species, it appears that host and pathogen genotypes contain multiple alleles that interact in a quantitative fashion to determine the level of resistance or susceptibility. Synthetic allopolyploids faithfully display additive virus resistance characteristics that correspond to modified matching allele interactions (Figure 4). Virus resistance/susceptibility factors change in allopolyploid progeny due to classical drift and selection (Figure 5). These changes raise the interesting possibility that moderately resistant allopolyploids could provide a hybrid bridge, which could result in a new Red Queen cycle of coevolution.

## Methods

### Ethics statement

This research did not involve research on human subjects, human material or human data, nor did it involve work with regulated invertebrates.

### Plant material

Seventeen *Nicotiana* species and seven synthetic allopolyploids (Table 1) were assayed for nepovirus resistance. With the exception of 2 × (*N. tabacum* × *N. benthamiana*), an infertile amphihaploid, all genetic materials were self-fertile. The synthetic allopolyploids exhibited no obvious phenotypic segregation. Seeds of 2 × (*N. tabacum* × *N. benthamiana*) [71] were a gift from Dr. G.B. Collins’s research program (University of Kentucky, Lexington, KY). Seeds of *N. benthamiana, N. tabacum* cv. Xanthi and *N. clevelandii* were from Drs. D. Gonsalves and R. Provvidenti (Cornell University New York State Agricultural Experiment Station, Geneva, NY). Seeds of *N. rustica*, *N. glauca, N. glutinosa* and *N. sylvestris* were obtained from commercial sources (Table 1). All of the other *Nicotiana* seeds were provided by the United States Nicotiana Germplasm Collection maintained at North Carolina State University (Raleigh, NC). Seedlings were grown in four-inch pots containing soilless potting media. Plants were grown in a greenhouse maintained at 24-26°C supplemented with high pressure sodium lamps for an 18 hour light/ 8 hour dark photoperiod, and watered daily or every other day as needed, and fertilized weekly.

### Virus strains and inoculation procedure

GFLV strain F13 from France [72,73] and strain GHu from Hungary [47,74] were isolated from infected grapevines, and ToRSV strain AP was isolated from an infected apricot tree in New York State [75]. GFLV and ToRSV strains were maintained in *N. benthamiana*. Virus inoculum was prepared by mechanically inoculating *N. benthamiana* and storing infected tissue at -80°C until inoculation of the host panel. Infected *N. benthamiana* tissue was ground 1:10 (w:v) in inoculation buffer (15 mM Na_2_HPO_4_ and 35 mM KH_2_PO_4_ pH 7.0) using a steel grinding set in a tissue lyser (Qiagen, Valencia, CA) and inoculated to three corundum-dusted leaves of each test plant with a ceramic pestle. Panels of four to 32 (median 17) plants per virus-host combination (Additional file 1: Table S1) were selected for uniformity in size and mechanically inoculated when they had 4–5 leaves and were approximately 3 cm in height. All plants were rinsed with water five to ten minutes after inoculation.

### Sampling and virus tests

Apical leaf positions were defined by counting nodes on the whorl upwards from the highest inoculated leaf. Apical leaves were collected at time points sufficient to detect cumulative virus infection: nine to 18 dpi for position one, 17 to 28 dpi for position two, and 26–60 dpi for position three. In plants where intermediate resistance phenotypes were observed, additional collections were made at 41 to 57 dpi for position four. Inoculated leaves were collected and processed between 21 and 54 dpi.

Plant tissue was collected from inoculated plants and processed for virus detection via double antibody-sandwich enzyme-linked immunosorbent assay (DAS-ELISA). Fresh tissue was ground in 1:10 (w:v) in 25 mM sodium phosphate buffer using a semi-automated HOMEX 6 tissue homogenizer and mesh grinding bags (Bioreba, Reinach, Switzerland). DAS-ELISAs for GFLV and ToRSV were carried out in Nunc MaxiSorp^®^ flat-bottom 96 well polystyrene microtiter plates (Fisher Scientific, Pittsburgh, PA) according to the manufacturer’s protocol (Bioreba). Absorbance (OD_405nm_) was measured after two hours of substrate incubation using a BioTek Synergy2 plate reader and Gen5 software was used to calculate blank-subtracted absorbance (Biotek, Winooski, VT). Each ELISA plate contained positive and negative checks, and the validity of each assay was ascertained before data was processed. Samples were considered positive if their absorbance values were greater than two times the mean absorbance values of negative controls.

### Evaluation of infection phenotypes

Virus symptoms were monitored daily on inoculated and apical plant leaves. Leaf samples that were positive or negative in DAS-ELISA for GFLV or ToRSV in each inoculation group were counted and converted into percent infection at each leaf position. Six resistance categories were assigned based on the infection outcome in inoculated leaves and in successive apical leaves. Virus-host combinations that yielded no detectable virus in the inoculated leaf (and apical leaves) were designated as ‘immune’ (category 1). ‘Early recovery’ (category 2) was defined as any level of inoculated leaf infection (10% to 100%) but the virus was rarely or infrequently (<10%) detected in the first apical leaf. ‘Late recovery’ (category 3) was defined at 10% to 100% infection in the first or second apical leaf position but a decline in virus incidence at higher leaf axes. ‘Intermediate recovery’ (category 4) was defined as 20% to 80% infection frequencies in all leaf axes, and no clear pattern of reduction or expansion of virus incidence in successively higher axes. ‘Delayed susceptibility’ (category 5) was defined as a steady increase in virus incidence at successively higher apical leaf axes until the highest tested position contained >75% frequency of virus infection. ‘Full susceptibility’ (category 6) was defined as virus incidence in 100% of apical leaves. The inoculated leaf was tested to discern immunity from early recovery.

### Tests for systemic recovery

A subset of the host panel exhibiting recovery from inoculation with GFLV strains F13 or GHu [4*x*(*N. sylvestris* × *N. tomentosiformis*), 4 × (*N. glutinosa* × *N. tabacum*) and 4 × (*N. sylvestris* × *N. otophora*)] was re-inoculated with GFLV-GHu in the fourth leaf position 34 days after the original inoculation. Re-inoculated leaves were tested for GFLV incidence at five dpi by DAS-ELISA. Negative values were interpreted as systemic recovery and positive values were interpreted as a lack of systemic recovery.

### Statistics

Statistics were computed on JMP version 10.0 (SAS Institute, Cary, NC). A score of one was assigned for each leaf infected in the first three apical leaf positions, and the sum of these scores among the samples at each leaf position is referred to as virus incidence. Each plant inoculated with a given virus was considered a replicate. Contingency analyses were used to compute Pearson’s correlations (*r*) and contingency tables. Correlation analyses were made for species origin (South America, California, Australia or synthetic), ploidy (*x* = 12 to 48), and virus inoculum (GFLV-F13, GFLV-GHu or ToRSV-AP) with respect to virus incidence at each leaf position. Origin and virus inoculum was considered as categorical variables, ploidy as continuous and virus incidence as ordinal data. A contingency analysis for section was not included due to the limited instances in which multiple species were sampled within a section. Correlation analyses were conducted where synthetic allopolyploids were either included or excluded in the data set.

## Competing interests

The authors declare they have no competing interests.

## Authors’ contributions

JG conceived the research, carried out virus inoculations and tests, analyzed the data and drafted the manuscript. SS and JG performed statistical analyses. RL, SS and MF critically revised the manuscript. RL provided much of the plant material and MF participated in the study design. All authors approved the final manuscript.

## Supplementary Material

Additional file 1: Table S1 Plant responses to *Grapevine fanleaf virus* (GFLV) strains F13 and GHu, and *Tomato ringspot virus* (ToRSV) strain AP. The data set supporting the results of the article is available in the Dryad Digital Repository in a Microsoft Word Document, doi:10.5061/dryad.3543v [76]. Click here for file
