# Supplementary material for: Allopolyploidy and the evolution of plant virus resistance
Source: BMC Evol Biol. 2014 Jul 3;14:149. doi: 10.1186/1471-2148-14-149 (PMC4226957; doi:10.1186/1471-2148-14-149)
Supplement: Additional file 1: Table S1 — Plant responses to Grapevine fanleaf virus (GFLV) strains F13 and GHu, and Tomato ringspot virus (ToRSV) strain AP. The data set supporting the results of the article is available in the Dryad Digital Repository in a Microsoft Word Document, doi:10.5061/dryad.3543v [76]. [file 1471-2148-14-149-S1.docx]

**Supplementary information**

**Table S1:** Plant responses to *Grapevine fanleaf virus* (GFLV) strains F13 and GHu, and *Tomato ringspot virus* (ToRSV) strain AP.

| Virus | Sample size^a^ | Species or  synthetic allopolyploid | Resistance  category^b^ | | Inoculated leaf^c,d^ | Apical leaf 1 | Apical leaf 2 | Apical leaf 3 | Apical leaf 4 |
| --- | --- | --- | --- | --- | --- | --- | --- | --- | --- |
| GFLV-F13 | 25 | 4*x*(*N. sylvestris* x *N. tomentosiformis*) | | 1 | 0% | 0% | N/T | N/T | N/T |
|  | 25 | 4*x*(*N. glutinosa* x *N. tabacum*) | | 1 | 0% | 0% | N/T | N/T | N/T |
|  | 19 | 4*x*(*N. sylvestris* x *N. otophora*) | | 1 | 0% | 0% | N/T | N/T | N/T |
|  | 6 | *N. paniculata* | | 1 | 0% | 0% | 0% | 0% | N/T |
|  | 10 | *N. tabacum* | | 1 or 2 | N/T | 0% | 0% | 0% | N/T |
|  | 30 | 4*x*(*N. rustica* x *N. tabacum*) | | 2 | 43% | 0% | N/T | N/T | N/T |
|  | 9 | *N. otophora* | | 2 | 44% | 0% | 0% | 0% | N/T |
|  | 6 | *N. tomentosiformis* | | 2 | 67% | 0% | 0% | 0% | N/T |
|  | 9 | *N. debneyi* | | 2 | 89% | 0% | 0% | 0% | N/T |
|  | 24 | 2*x*(*N. tabacum* x *N.*  *benthamiana*) | | 3 | 69% | 50% | 4% | 0% | N/T |
|  | 16 | 4*x*(*N. quadrivalvis* x *N. tabacum*) | | 3 | 100% | 25% | 19% | 6% | N/T |
|  | 30 | 4*x*(*N. debneyi* x *N. clevelandii*) | | 4 | N/T | 70% | 70% | 67% | N/T |
|  | 24 | *N. clevelandii* | | 6 | 100% | 100% | 100% | 100% | N/T |
|  | 24 | *N. benthamiana* | | 6 | 100% | 100% | 100% | 100% | N/T |
| GFLV-GHu | 14 | *N. obtusifolia* | | 2 | 13% | 0% | 0% | 0% | 0% |
|  | 7 | *N. glauca* | | 2 | 14% | 0% | 0% | 0% | 0% |
|  | 13 | *N. sylvestris* | | 2 | 54% | 0% | 0% | 8% | 0% |
|  | 4 | *N. kawakamii* | | 2 | 75% | 0% | 0% | 0% | N/T |
|  | 23 | *N. tabacum* | | 2 | 78% | 0% | 4% | 0% | 0% |
|  | 10 | *N. tomentosiformis* | | 2 | 100% | 0% | 10% | 10% | 0% |
|  | 32 | 4*x*(*N. sylvestris* x *N. tomentosiformis*) | | 3 | 50% | 16% | 3% | 3% | 0% |
|  | 30 | 4*x*(*N. quadrivalvis* x *N. tabacum*) | | 3 | 57% | 10% | 10% | 3% | N/T |
|  | 10 | *N. paniculata* | | 3 | 60% | 10% | 30% | 0% | 0% |
|  | 4 | *N. setchelii* | | 3 | 75% | 25% | 0% | 0% | N/T |
|  | 21 | 4*x*(*N. sylvestris* x *N. otophora*) | | 3 | 81% | 10% | 5% | 0% | N/T |
|  | 8 | *N. glutinosa* | | 3 | 100% | 13% | 0% | 0% | N/T |
|  | 24 | 4*x*(*N. glutinosa* x *N. tabacum*) | | 3 | 100% | 12% | 4% | N/T | N/T |
|  | 30 | 2*x*(*N. tabacum* x *N. benthamiana*) | | 4 | 80% | 50% | 57% | 60% | 53% |
|  | 14 | 4*x*(*N. rustica* x *N. tabacum*) | | 4 | 100% | 33% | 24% | 36% | N/T |
|  | 5 | *N. rustica* | | 4 | 100% | 80% | 40% | 60% | 20% |
|  | 8 | *N. debneyi* | | 4 | 100% | 33% | 89% | 89% | 88% |
|  | 7 | *N. otophora* | | 5 | 100% | 0% | 56% | 44% | 78% |
|  | 24 | *N. suaveolens* | | 5 | N/T | 4% | 83% | 79% | N/T |
|  | 19 | 4*x*(*N. debneyi* x *N. clevelandii*) | | 5 | N/T | 93% | 93% | 100% | N/T |
|  | 8 | *N. attenuata* | | 6 | 100% | 100% | 100% | 100% | N/T |
|  | 24 | *N. clevelandii* | | 6 | 100% | 100% | 100% | 100% | N/T |
|  | 24 | *N. benthamiana* | | 6 | 100% | 100% | 100% | 100% | N/T |
|  | 12 | *N. goodspeedii* | | 6 | N/T | 100% | 100% | N/T | N/T |
| ToRSV-AP | 8 | *N. otophora* | | 3 | N/T | 100% | 0% | 0% | N/T |
|  | 16 | *N. tabacum* | | 3 | N/T | 88% | 19% | 6% | N/T |
|  | 16 | *N. setchelii* | | 3 | N/T | 100% | 13% | 0% | N/T |
|  | 11 | *N. kawakamii* | | 3 | N/T | 100% | 27% | 0% | N/T |
|  | 12 | 4*x*(*N. rustica* x *N. tabacum*) | | 3 | N/T | 100% | 100% | 0 | N/T |
|  | 23 | 4*x*(*N. sylvestris* x *N. tomentosiformis*) | | 3 | N/T | 100% | 83% | 0% | N/T |
|  | 27 | 4*x*(*N. sylvestris* x *N. otophora*) | | 3 | N/T | 100% | 89% | 26% | N/T |
|  | 16 | 2*x*(*N. tabacum* x *N. benthamiana*) | | 5 | N/T | 75% | 81% | 100% | N/T |
|  | 12 | 4*x*(*N. quadrivalvis* x *N. tabacum*) | | 6 | N/T | 100% | 100% | 100% | N/T |
|  | 24 | *N. benthamiana* | | 6 | N/T | 100% | 100% | 100% | N/T |

^a^The sample size denotes the lowest number of samples tested at any given time point to account for missing samples.

^b^Categories of resistance (1, most resistant, through 6, most susceptible) are indicated for each virus-host combination tested.

^c^Values represent the percent of plants in the sample showing detectable virus at each given leaf position.

^d^N/T: Not tested
